# Retinal vascular alterations as assessment indicators of atherosclerotic cardiovascular disease risk in dyslipidemia patients

**DOI:** 10.3389/fcvm.2025.1634816

**Published:** 2025-09-12

**Authors:** Yu He, Guo-hong Wang, Ming-zhao Qin, Kai Cao, Yong-peng Zhang, Xuan Jiao, Zheng Zhang, Qi Liu, Qian Liu, Jin-bao Ma

**Affiliations:** ^1^ Department of Geriatrics, Beijing Tongren Hospital, Capital Medical University, Beijing, China; ^2^ Beijing Institute of Ophthalmology, Beijing Tongren Hospital, Capital Medical University, Beijing, China; ^3^ Department of Ophthalmology, Beijing Tongren Hospital, Capital Medical University, Beijing, China

**Keywords:** dyslipidemia, ASCVD, risk, biomarkers, retinal vessels, opticalcoherence tomography angiography

## Abstract

**Introduction:**

Atherosclerotic cardiovascular disease (ASCVD) remains the leading cause of global mortality, particularly among individuals with dyslipidemia. Traditionally, the retina has been considered a key site for examining microvascular changes. Recent evidence, however, indicates that retinal alterations may also reflect macrovascular changes. This study proposes a hypothesis in which Optical Coherence Tomography Angiography (OCTA) is utilized to evaluate retinal vascular changes as a potential biomarker for ASCVD risk assessment in dyslipidemia patients.

**Methods:**

In this cross-sectional study, 261 dyslipidemia patients were recruited and classified into non-ASCVD and ASCVD groups. OCTA was performed on all patients, with the macula and optic disc being the primary areas of assessment. The following parameters were measured: retinal vessel density (VD), retinal nerve fiber layer (RNFL) thickness, retinal thickness, foveal avascular zone (FAZ) area, FAZ perimeter, and VD within a 300 μm width ring surrounding the FAZ (FD). Ultimately, data from 231 eyes were analyzed. Comparisons of OCTA-derived metrics between groups were made, and receiver operating characteristic (ROC) curve analysis was conducted to assess the discriminatory power of these metrics for identifying ASCVD in dyslipidemia patients. The DeLong test was used to compare areas under the ROC curve for these indicators. All statistical tests were two-tailed, with significance set at *P* *<* 0.05.

**Results:**

In the ASCVD group, RNFL thickness, superficial capillary plexus (SCP) parafoveal VD, SCP perifoveal VD, macular parafoveal thickness, and FD were significantly lower compared to the non-ASCVD group. ROC curve analysis confirmed the predictive value of these indicators for ASCVD identification in dyslipidemia patients. SCP parafoveal VD, SCP perifoveal VD, and FD correspond to the macular superficial capillary plexus vessel densities. When combined, these indicators formed a new composite measure, macular superficial vessel density (MSVD). The ROC curve further validated MSVD's predictive utility for ASCVD in dyslipidemia patients, with the optimal threshold identified at 143.22% using the Youden index.

**Conclusions:**

OCTA-derived indicators, particularly MSVD, demonstrate significant potential as novel biomarkers for ASCVD risk assessment in dyslipidemia patients.

## 1 Introduction

Atherosclerotic cardiovascular disease (ASCVD), including coronary atherosclerotic heart disease (CHD), ischemic stroke, and peripheral arterial disease, remains the leading global cause of mortality (1–3). CHD can result in myocardial infarction and sudden cardiac death, ischemic stroke can lead to aphasia, prolonged immobility, and secondary cerebral hemorrhage, and peripheral arterial disease in the lower extremities may cause impaired mobility or necessitate amputation (4–7). These conditions severely impair patients’ quality of life, impose significant economic burdens, and increase mortality rates. Accurate risk stratification and early identification of individuals at high risk are essential to prevent disease initiation, mitigate progression, and reduce associated mortality.

The pathophysiological hallmark of ASCVD is the formation and advancement of atherosclerotic plaques, with dyslipidemia, particularly elevated low-density lipoprotein cholesterol (LDL-C), recognized as a principal risk factor (1–3). Evaluating ASCVD risk in individuals with dyslipidemia, predominantly characterized by elevated LDL-C levels, is crucial for implementing targeted interventions and enhancing clinical outcomes.

Previous ASCVD risk assessment tools have been developed (8–11), but they do not specifically cater to the needs of high-risk individuals with dyslipidemia. The predictive variables used, such as blood pressure, lipid levels, and waist circumference, exhibit considerable fluctuation. This highlights the importance of identifying stable, reliable biomarkers for risk evaluation. The retina, recognized as a window into microvascular health, has gained attention as a potential indicator for coronary artery alterations. Recent studies reveal significant differences in retinal vascular features between CHD patients and controls, with these differences correlating with the extent of coronary artery stenosis (12, 13). This evidence suggests an association between retinal microvascular changes and macrovascular conditions. Thus, the current study investigates the feasibility of assessing ASCVD risk in individuals with dyslipidemia by analyzing retinal vascular characteristics.

Optical coherence tomography angiography (OCTA) represents an advanced, non-invasive imaging technique that enables rapid and quantitative evaluation of retinal vascularity (14). Compared to traditional methods, such as color fundus photography and fundus fluorescein angiography, OCTA offers clear advantages. The former lacks quantitative capabilities, while the latter necessitates the injection of a contrast agent, making it invasive. OCTA provides key metrics, including retinal vascular density (VD), retinal nerve fiber layer (RNFL) thickness, and foveal avascular zone (FAZ) area (15), which together allow for a more thorough examination of retinal microvasculature. Previous investigations utilizing OCTA to assess retinal vascular changes have demonstrated significant alterations in the fundus vessels of patients with hypertension (16), diabetes (17), and dyslipidemia (18) when compared to controls. These conditions are also recognized as risk factors for ASCVD. The aim of this study is to investigate the role of retinal vascular alterations assessed by OCTA as biomarkers for ASCVD risk assessment in dyslipidemia patients.

## 2 Materials and methods

### 2.1 Study design and allocation

The study conformed to the ethical guidelines set forth in the *Declaration of Helsinki* and was approved by the Ethics Committee of Beijing Tongren Hospital, Affiliated to Capital Medical University (approval No. TRECKY2021-227). Written informed consent was obtained from all participants or their legal representatives. The trial was registered under NCT05397158.

A cross-sectional design was employed, enrolling dyslipidemia patients admitted to the Department of Geriatrics, Beijing Tongren Hospital, between December 2022 and March 2024. Inclusion criteria required participants to be aged ≥18 years, have a history or confirmed diagnosis of dyslipidemia during hospitalization, and consent to OCTA examination. Exclusion criteria included glaucoma, inability to comply with OCTA procedures, severe cataract or fundus hemorrhage obstructing OCTA imaging, acute infections, malignancies, active autoimmune diseases, or acute myocardial infarction. Dyslipidemia was defined as LDL-C exceeding target treatment levels according to risk stratification (1–3) or a history of dyslipidemia with prolonged statin and/or proprotein convertase subtilisin/kexin type 9(PCSK9) inhibitor use. Participants were categorized into two groups based on their medical history and hospitalization results: those without ASCVD and those diagnosed with ASCVD. ASCVD was defined as including CHD, ischemic stroke, and peripheral arterial disease (1, 2). CHD included a documented history of acute coronary syndrome (e.g., myocardial infarction or unstable angina), stable angina, or coronary revascularization procedures, such as percutaneous coronary intervention, coronary artery bypass grafting, or other arterial interventions (4, 5). Stroke classification was confined to ischemic stroke (6), while peripheral arterial disease included lower-extremity arterial disease and carotid artery stenosis (7).

### 2.2 Data collection

Patient data were extracted from the hospital's medical record system. Collected information included demographic variables (sex, age, smoking history, height, and weight), medical conditions [hypertension, diabetes, hyperuricemia, obstructive sleep apnea hypopnea syndrome (OSAHS), CHD, stroke, and peripheral arterial disease], current treatments (antihypertensives, hypoglycemic agents, statins, PCSK9 inhibitors, antiplatelet agents, and anticoagulants), and laboratory results (total cholesterol (TC), LDL-C, high-density lipoprotein cholesterol (HDL-C), triglycerides (TG), lipoprotein(a) [Lp(a)], serum creatinine, cystatin C, glycosylated hemoglobin (HbA1c), and estimated glomerular filtration rate (eGFR) calculated using the CKD-EPI (creatinine-cystatin C equation) (19). Furthermore, 24-hour ambulatory blood pressure (ABP) readings, including mean systolic blood pressure (MSBP) and mean diastolic blood pressure (MDBP), coronary angiography or coronary computed tomography angiography (CCTA) results, and peripheral artery ultrasound findings (carotid and lower extremity arteries) were incorporated.

### 2.3 OCTA

OCTA imaging was conducted on 6 × 6 mm angiograms using the RTVue XR system with Avanti software version 2017.1.0.155 (Optovue, Inc., Fremont, CA, USA). The system's parameters included an image acquisition rate of 70,000 A-scans/second, an axial resolution of 5 μm, and a scan depth range of 2–3 mm, with a transverse range of 2–12 mm. The scanning beam operated at a wavelength of 840 ± 10 nm, spanning from the internal limiting membrane to the retinal pigment epithelium. Images were deemed acceptable if the signal intensity was ≥6. Mydriasis was induced in patients using tropicamide 30 minutes prior to the OCTA examination. The procedures were performed by trained ophthalmic technicians, with data collected from the right eye of each participant (20). For the four participants with functional vision exclusively in the left eye, data from the left eye were included in the analysis.

The retina's key anatomical landmarks include the optic disc and macula (21). The optic disc serves as the point of entry for the central retinal artery, the point of exit for the retinal vein, and the conduit for the optic nerve head, from which the optic nerve emerges. The macula is the region with the highest density of photoreceptor cells. Retinal blood flow is supplied by the central retinal artery and its branching capillaries, which connect to venous branches. The central retinal artery enters through the optic disc, dividing into smaller capillaries. Within the macula, these capillaries narrow, forming a circular-shaped structure devoid of vessels at its center, known as the FAZ.

**Figure 1:**
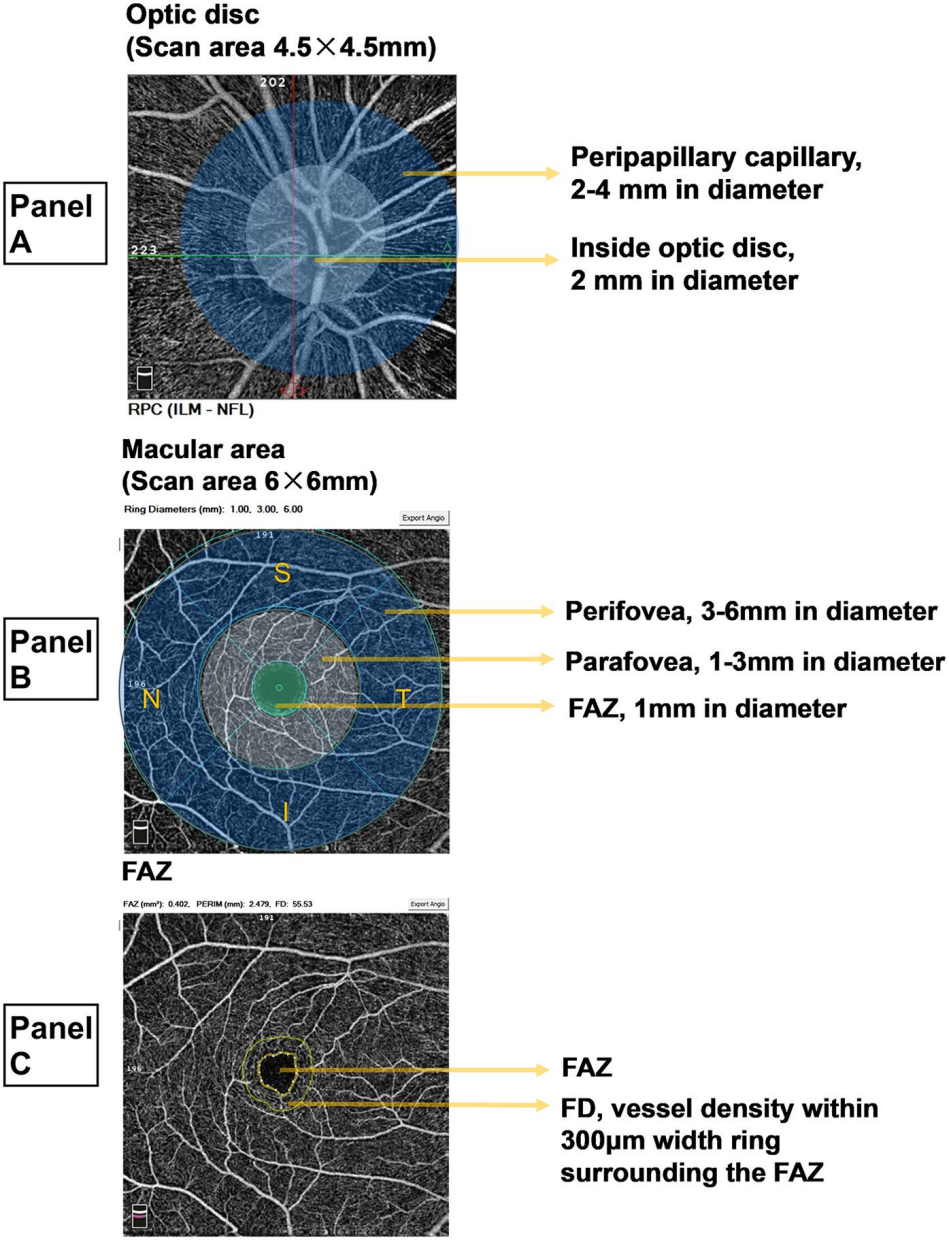
Measurement zones and parameters for OCTA. **(A)** Optic disc area. The radial peripapillary capillary (RPC) region in the optic disc area encompasses the optic disc (a circular area with a 2-mm diameter) and the peripapillary capillary zone (a ring-shaped area with a diameter of 2-4 mm). The RPC vascular density (VD) is calculated as the mean VD of all vessels across the image and the mean VD of all small vessels. The retinal nerve fiber layer (RNFL) thickness is measured as the average value within the peripapillary capillary region. **(B)** Macular region. The macular region is analyzed by dividing it into two capillary plexus layers: the superficial capillary plexus (SCP) and the deep capillary plexus (DCP). Both layers share identical measurement divisions, as illustrated using the SCP. The perifoveal region, represented by the outer ring, has a diameter of 3–6 mm, while the parafoveal region, denoted by the inner ring, has a diameter of 1-3 mm. VD measurements within these rings represent the mean values for four quadrants: superior (S), inferior (I), temporal (T), and nasal (N). At the center, the foveal avascular zone (FAZ) is a circular area with a 1-mm diameter. VD is expressed as a percentage, indicating the proportion of vascularized area within the measurement zone. **(C)** FAZ. The FAZ represents the foveal avascular zone. The FAZ constitutes the central avascular circular zone, while the surrounding ring corresponds to the VD within 300 μm width ring surrounding the FAZ (FD).

OCTA facilitates the evaluation of various retinal parameters, including VD in the optic disc region, RNFL thickness, VD and thickness in the macular region, FAZ area and perimeter, and VD within a 300 μm ring surrounding the FAZ (FD) (Figure 1). The retinal vasculature in the optic disc region is organized radially, forming the radial peripapillary capillary (RPC) network, which is divided into two zones: the inside disc and the peripapillary capillary areas. OCTA provides average VD values for both total and small blood vessels within these zones, while RNFL thickness measurements reflect the average in the peripapillary capillary region. In the macular region, the vasculature is categorized into two layers: the superficial capillary plexus (SCP) and the deep capillary plexus (DCP). SCP, extending from the internal limiting membrane to the inner plexiform layer, demonstrates uniform blood flow and regular contours. DCP, located between the inner and outer plexiform layers, consists of finer vasculature with similarly regular morphology. VD measurements are taken within two concentric zones: the parafovea (1–3 mm diameter) and perifovea (3–6 mm diameter). The calculated VD represents the mean value across four quadrants: superior (S), inferior (I), temporal (T), and nasal (N). VD is expressed as a percentage, reflecting the proportion of the area occupied by blood vessels, without distinguishing between arteries and veins. Utilizing the AngioAnalytics license, the OCTA system visualizes retinal VD parameters through color-coded mapping, with distinct colors representing specific VD ranges (e.g., green indicating a VD of 45%–55%). Retinal VD in healthy individuals is solely associated with age (22). Standard RNFL thickness measures approximately 100 μm (23), while macular thickness typically ranges from 200 to 300 μm (14). In contrast, the FAZ area shows considerable variability in healthy individuals with normal vision, ranging from 0.071 mm² to 0.527 mm² (24).

Color fundus photography and OCTA examinations were conducted for all patients. An ophthalmologist provided consultation to explain the ophthalmic findings to the patients. As the most commonly employed fundus imaging technique in clinical settings, color fundus photography assists in detecting retinal exudation, hemorrhage, and neovascularization; however, it lacks the ability for quantitative analysis (14). Consequently, the results obtained from color fundus photography in this study served merely as a reference for ophthalmologists during consultations and were not subjected to quantitative evaluation.

### 2.4 Statistical analysis

Quantitative variables were expressed as mean ± standard deviation for normally distributed data and as median with interquartile range for non-normally distributed data, while qualitative variables were presented as frequencies with percentages. The normality of quantitative data was assessed before analysis. *T*-tests were applied to compare normally distributed data, whereas non-normally distributed data were evaluated using rank-sum tests. Chi-square tests were utilized for qualitative data analysis. Receiver operating characteristic (ROC) curves were generated to assess the discriminative ability of RNFL thickness, SCP parafovea VD, SCP perifovea VD, macular parafovea thickness, and FD in identifying ASCVD. The areas under the ROC curve (AUC) for these indicators were compared using the DeLong test. The optimal threshold was determined using the Youden index, calculated as sensitivity plus specificity minus one, with the threshold corresponding to the highest Youden index designated as optimal. All statistical tests were two-tailed, with significance set at *P* *<* 0.05. Analyses were performed using SPSS 29.0 software (SPSS, Inc., Chicago, IL, USA).

This study examined the relationship between OCTA-derived indices and ASCVD. Previous studies investigating the association between retinal vascular parameters and coronary artery disease typically included sample sizes ranging from 200 to 300 patients. The current study adhered to this sample size, fulfilling the requirements for intergroup comparisons (*t*-test, rank-sum test) and ROC curve analysis.

## 3 Results

**Figure 2:**
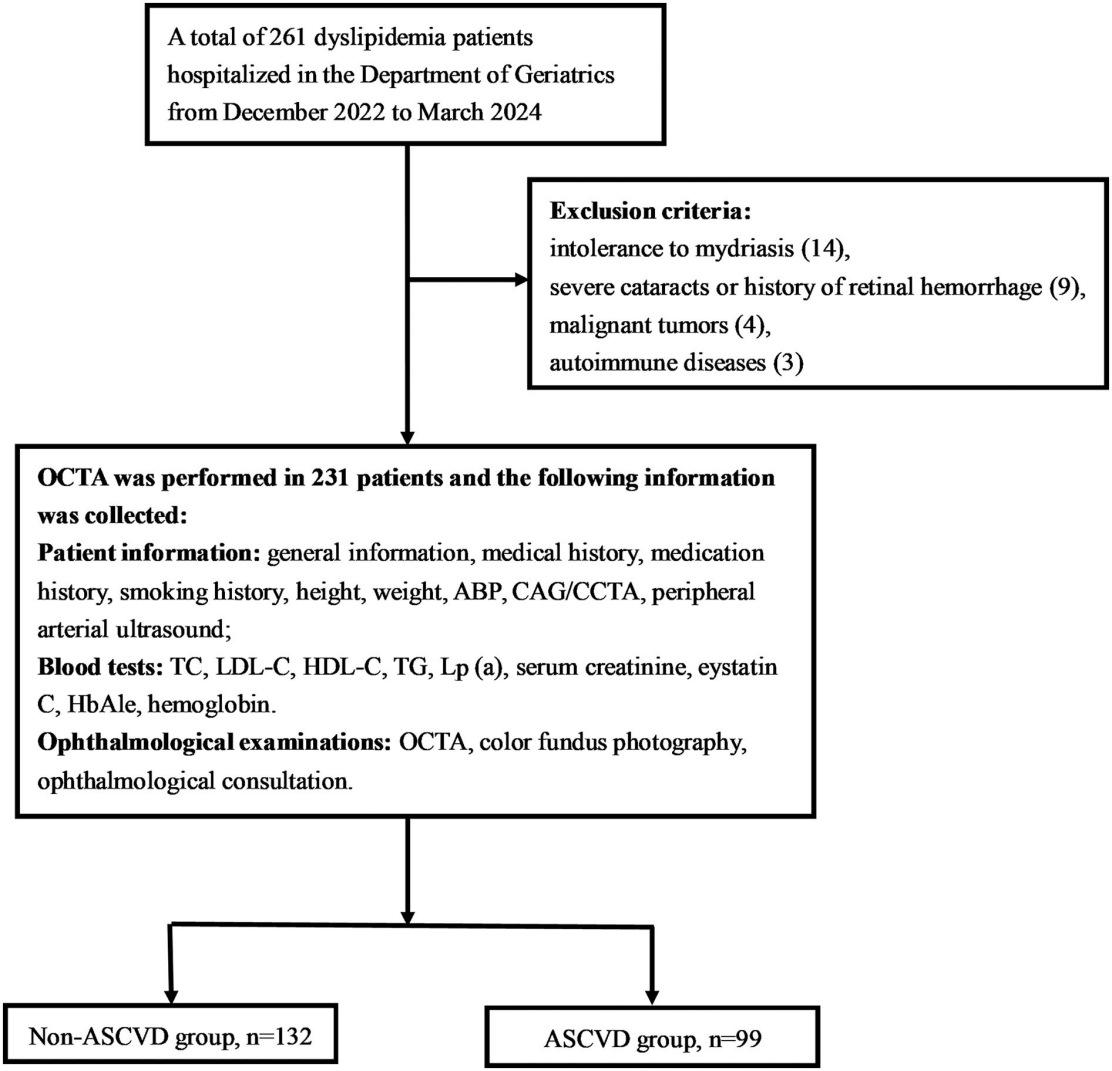
Flow chart. OCTA, optical coherence tomography angiography; ABP, ambulatory blood pressure; CAG, coronary angiography; CCTA, coronary computed tomography angiography; TC, total cholesterol; LDL-C, low-density lipoprotein cholesterol; HDL-C, high-density lipoprotein Cholesterol; TG, triglyceride; Lp(a), lipoprotein(a).

A total of 261 dyslipidemic patients were initially assessed for eligibility in this study, all of whom were of yellow races. Fourteen patients were excluded due to intolerance to mydriasis, preventing OCTA examination; nine were excluded for having severe cataracts or a history of fundus hemorrhage; four had malignant tumors; and three were in the active phase of autoimmune diseases. Consequently, the final cohort consisted of 231 participants (458 eyes in total), including 170 males (73.6%) with a mean age of 63.68 ± 11.61 years (Figure 2).

Participants were classified into two groups based on the presence or absence of ASCVD: the non-ASCVD group (*n* = 132) and the ASCVD group (*n* = 99). A comparison of baseline characteristics between the groups is provided in Table 1. The ASCVD group was older and had a higher incidence of hypertension, increased use of antihypertensive medications, hypoglycemic agents, statins or PCSK9 inhibitors, antiplatelet agents, and anticoagulants, alongside elevated MSBP and HbA1c levels. Conversely, the non-ASCVD group exhibited higher levels of TC, LDL-C, and eGFR. No significant differences were noted between the groups concerning sex, diabetes, hyperuricemia, OSAHS, smoking status, BMI, MDBP, HDL-C, TG, Lp(a), or hemoglobin.

**Table 1:** Baseline demographics and clinical characteristics.

	non-ASCVD (*n* = 132)	ASCVD (*n* = 99)	*P* value
Age (years), mean ± SD	60.28 ± 11.92	68.21 ± 9.49	<0.001
Sex (male), *n* (%)	94 (71.2%)	76 (76.8%)	0.343
Comorbidity			
Hypertension, *n* (%)	84 (63.6%)	84 (84.8%)	<0.001
Diabetes mellitus, *n* (%)	72 (54.5%)	64 (64.6%)	0.123
Hyperuricemia, *n* (%)	24 (18.2%)	19 (19.2%)	0.845
OSAHS, *n* (%)	17 (12.9%)	15 (15.2%)	0.621
Medication			
Antihypertensive drugs, *n* (%)	61 (46.2%)	78 (78.8%)	<0.001
Hypoglycemic agents, *n* (%)	41 (31.1%)	58 (58.6%)	<0.001
Statin and/or PCSK9 inhibitors, *n* (%)	56 (42.4%)	75 (75.8%)	<0.001
Antiplatelet agents, *n* (%)	15 (11.4%)	66 (66.7%)	<0.001
Anticoagulants, *n* (%)	1 (0.8%)	6 (6.1%)	0.020
Smoking and ex-smoking, *n* (%)	65 (49.2%)	56 (56.6%)	0.270
BMI (kg/m^2^), mean ± SD	25.18 ± 3.34	25.45 ± 2.85	0.519
Mean systolic blood pressure (mmHg), median [Q1; Q3]	122.5[117; 134.75]	129[120;142]	0.011
Mean diastolic blood pressure (mmHg), median [Q1; Q3]	72[69; 78.75]	73[66;80]	0.909
Laboratory test			
TC, median [Q1; Q3]	4.72[3.88;5.38]	3.71[3.22;4.66]	<0.001
LDL-C (mmol/L), median [Q1; Q3]	2.75[2.14;3.43]	1.98[1.62;2.94]	<0.001
HDL-C, median [Q1; Q3]	1.16[0.95;1.38]	1.11[0.93;1.38]	0.428
TG, median [Q1; Q3]	1.43[0.95;2.1]	1.38[0.91;1.93]	0.495
Lp(a), median [Q1; Q3]	9.3[3.88;23.23]	8.7[4.1;34.4]	0.621
HBAIC (%), median [Q1; Q3]	6.20[5.73;6.83]	6.7[6;7.6]	0.010
Hemoglobin (g/L), mean ± SD	138.70 ± 15.46	134.95 ± 14.27	0.060
eGFR (ml/min), median [Q1; Q3]	97.43[82.59;107.26]	80.06[67.6;96.68]	<0.001

ASCVD, arteriosclerotic cardiovascular disease; SD, standard deviation; OSAHS, obstructive sleep apnea hypopnea syndrome; BMI, body mass index; Q1, lower quartile; Q3, upper quartile; TC, total cholesterol; LDL-C, low-density lipoprotein cholesterol; HDL-C, high-density lipoprotein cholesterol; TG, Triglyceride; Lp(a), Lipoprotein a; HbA1c, glycated hemoglobin; eGFR, estimated glomerular filtration rate.

In the optic disc region, the ASCVD group exhibited a reduced RNFL thickness compared to the non-ASCVD group [111.50[102.36;118.61] vs. 107.00[97.00;118.00], *P* *=* 0.040]. No significant differences were found in the VD of total or small vessels between the two groups in this region (Table 2).

**Table 2:** Comparison of OCTA results.

	non-ASCVD	ASCVD	*P* value
	(*n* = 132)	(*n* = 99)	
Optic disc			
RNFL thickness (μm), median [Q1; Q3]	111.50[102.36;118.61]	107.00[97.00;118.00]	0.040
RPC total VD (%), median [Q1; Q3]	54.44[52.84;56.78]	54.00[50.00;56.80]	0.179
RPC total small VD (%), median [Q1; Q3]	48.13[46.80;50.28]	47.96[45.10;50.40]	0.211
Macula			
SCP parafovea VD (%), median [Q1; Q3]	48.05[44.53;51.30]	45.32[41.90;49.10]	0.001
SCP perifovea VD (%), median [Q1; Q3]	47.40[43.53;49.92]	45.20[42.20;47.70]	0.001
DCP parafovea VD (%), median [Q1; Q3]	50.25[47.43;53.20]	49.90[47.40;53.10]	0.944
DCP perifovea VD (%), mean ± SD	43.94 ± 5.51	43.83 ± 5.36	0.885
Parafovea thickness (μm), median [Q1; Q3]	321.88[309.56;332.44]	316.50[303.75;329.25]	0.028
Perifovea thickness (μm), median [Q1; Q3]	281.25[272.88;286.69]	275.50[265.00;286.25]	0.052
FAZ			
Area (mm^2^), median [Q1; Q3]	0.29[0.23;0.38]	0.31[0.24;0.38]	0.722
Perimeter (mm), mean ± SD	2.17 ± 0.37	2.20 ± 0.44	0.613
FD (%), median [Q1; Q3]	49.63[45.40;53.31]	47.59[43.13;50.74]	0.003
Thickness (μm), median [Q1; Q3]	252.00[236.00;265.00]	251.00[236.00;268.00]	0.651

OCTA, optical coherence tomography angiography; ASCVD, arteriosclerotic cardiovascular disease; RNFL, retinal nerve fiber layer; Q1, lower quartile; Q3, upper quartile; RPC, radial peripapillary capillary; VD, vessel density; SCP, superficial capillary plexus; DCP, deep capillary plexus; SD, standard deviation; FAZ, foveal avascular zone; FD, VD within 300 μm width ring surrounding the FAZ.

**Figure 3:**
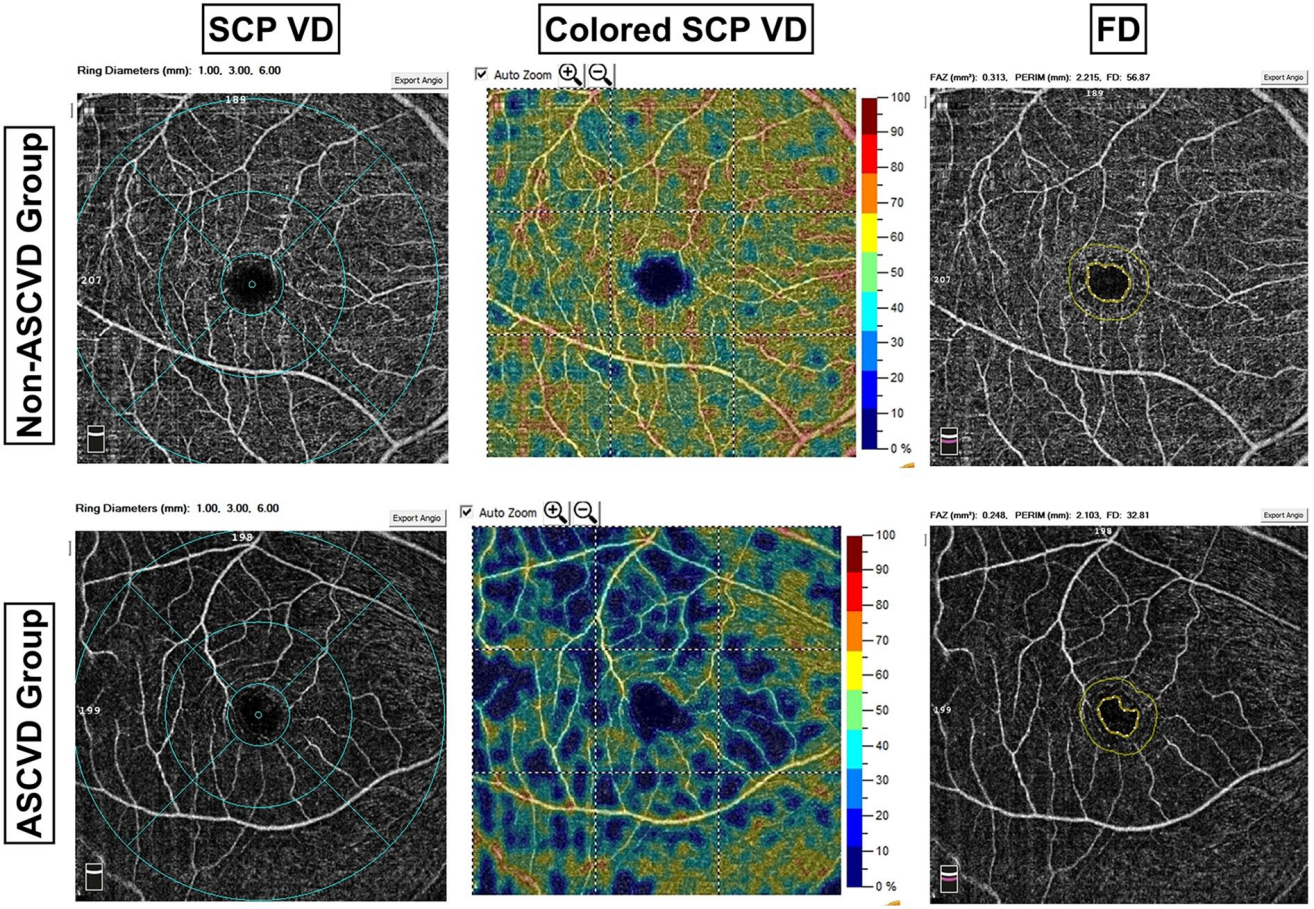
Comparison of OCTA images between groups. ASCVD, arteriosclerotic cardiovascular disease; SCP, superficial capillary plexus; VD, vessel density; FD, VD within 300 μm width ring surrounding the FAZ. The first column illustrates a comparison of SCP VD between the two groups, revealing sparser retinal vessels in the ASCVD group compared to the non-ASCVD group. The OCTA system employed in this study provides a color-coded visualization of retinal VD, as depicted in the second column. Each color corresponds to a specific VD range, with green representing 45%–55% VD. The ASCVD group exhibited a higher prevalence of blue regions and fewer green regions compared to the non-ASCVD group.

In the macular region, VD of the parafovea [48.05[44.53;51.30] vs. 45.32[41.90;49.10]*, P* *=* 0.001] and perifovea [47.40[43.53;49.92] vs. 45.20[42.20;47.70]*, P* *=* 0.001] in the SCP was significantly lower in the ASCVD group. OCTA imaging revealed a decrease in retinal vasculature density in the ASCVD group, confirmed by processed color images showing more extensive blue areas and fewer green areas (Figure 3). No significant difference was found in the VD of the parafovea [50.25[47.43;53.20] vs. 49.90[47.40;53.10], *P* *=* 0.944] or perifovea (43.94 ± 5.51 vs. 43.83 ± 5.36, *P* *=* 0.885) within the DCP between the two groups. Retinal thickness in the parafovea (321.88[309.56;332.44] vs. 316.50[303.75;329.25], *P* *=* 0.028) and perifovea [281.25[272.88;286.69] vs. 275.50[265.00;286.25], *P* *=* 0.052] was reduced in the ASCVD group, with only the parafoveal thickness difference reaching statistical significance (Table 2).

In the FAZ, no significant differences were observed between the groups in area, perimeter, or retinal thickness. However, the FD was significantly lower in the ASCVD group compared to the non-ASCVD group [49.63[45.40;53.31] vs. 47.59[43.13;50.74], *P* *=* 0.003], which was confirmed by statistical analysis (Table 2). Imaging also showed a decrease in FD in the ASCVD group, further supporting this finding (Figure 3).

**Figure 4:**
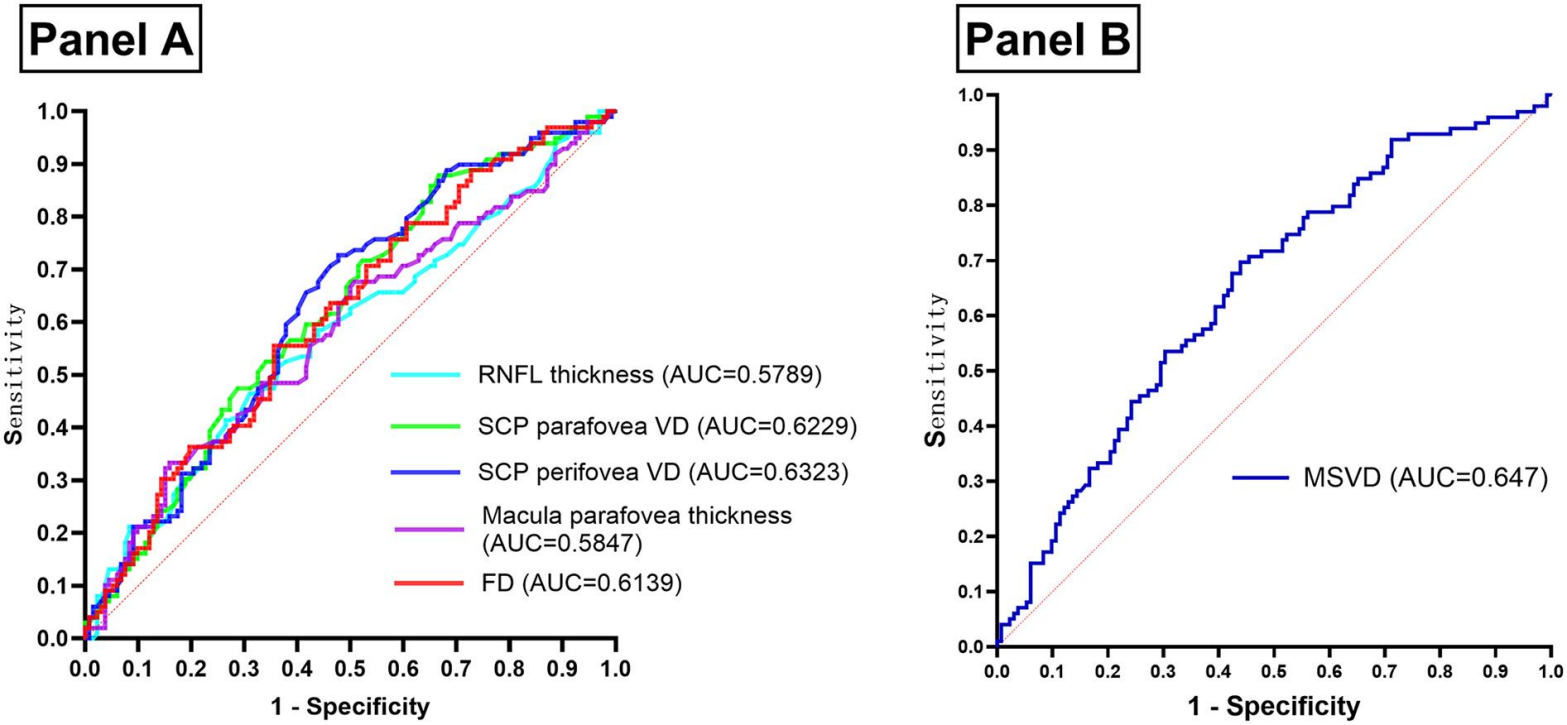
(Panel **A**) ROC curve of OCTA results assessing the occurrence of ASCVD in patients with dyslipidemia. (Panel **B**) ROC curve of MSVD assessing the occurrence of ASCVD in patients with dyslipidemia. OCTA, optical coherence tomography angiography; ASCVD, arteriosclerotic cardiovascular disease; ROC, receiver operating characteristic; AUC, area under curve; RNFL, retinal nerve fiber layer; SCP, superficial capillary plexus; VD, vessel density; FD, VD within 300 μm width ring surrounding the FAZ; MSVD, macula superficial vessel density.

A comparison of OCTA results between the two groups identified RNFL thickness, SCP parafovea VD, SCP perifovea VD, macular parafovea thickness, and FD as key parameters for constructing an ROC curve to evaluate their predictive value for ASCVD in dyslipidemia patients (Table 3 and Figure 4A). The analysis confirmed that all five parameters were indicative of ASCVD risk in this population. The optimal thresholds, determined via the Youden index, were found to be 47.275% for SCP perifovea VD, 50.850% for SCP parafovea VD, and 48.045% for FD.

**Table 3:** ROC curve parameters for OCTA results assessing ASCVD risk.

	AUC	95%CI	*P* value	Youden's index	Threshold
RNFL thickness (μm)	0.5789	0.5037–0.6541	0.0403	0.154	105.475
SCP parafovea VD (%)	0.6229	0.5509–0.6950	0.0014	0.212	50.850
SCP perifovea VD (%)	0.6323	0.5607–0.7040	0.0006	0.250	47.275
Parafovea thickness (μm)	0.5847	0.5097–0.6597	0.0276	0.174	307.375
FD (%)	0.6139	0.5413–0.6865	0.0031	0.199	48.045

OCTA, optical coherence tomography angiography; ASCVD, arteriosclerotic cardiovascular disease; ROC, receiver operating characteristic; AUC, area under curve; CI, confidence interval; RNFL, retinal nerve fiber layer; SCP, superficial capillary plexus; VD, vessel density; FD, VD within 300 μm width ring surrounding the FAZ.

Anatomically, the macular superficial layer, extending from medial to lateral, comprises the FAZ, SCP parafovea, and SCP perifovea, all of which are interconnected vascular regions (Figure 1B). Given the relatively intact terminal vascular ring of the superficial FAZ, FD is defined as the VD within a 300 μm-wide ring surrounding the superficial FAZ. As such, FD, along with SCP parafovea VD and SCP perifovea VD, serves as an indicator of superficial retinal VD. These three parameters were consolidated into a composite measure—macular superficial vessel density (MSVD)—to assess ASCVD risk in dyslipidemia patients. ROC curve analysis revealed an AUC of 0.647 for MSVD (95% CI: 0.576–0.718), which surpassed the AUCs for FD, SCP parafovea VD, and SCP perifovea VD. The maximum Youden index value of 0.258 indicated an optimal threshold of 143.22% (Figure 4B). DeLong test results indicated no statistically significant differences in AUC values when comparing MSVD with FD, SCP parafovea VD, SCP perifovea VD, RNFL thickness, and macular parafovea thickness (Table 4).

**Table 4:** Delong test of AUCs between MSVD and other indicators.

	Difference in AUC	95%CI	*P* value
MSVD—RNFL thickness	0.068	−0.015–0.152	0.109
MSVD—Parafovea thickness	0.062	−0.022–0.147	0.147
MSVD—SCP parafovea VD	0.024	−0.007–0.055	0.126
MSVD—SCP perifovea VD	0.015	−0.027–0.057	0.490
MSVD—FD	0.033	−0.012–0.078	0.145
RNFL thickness—Parafovea thickness	−0.006	−0.100–0.088	0.904

MSVD, macula superficial vessel density; ROC, receiver operating characteristic; AUC, area under the curve; RNFL, retinal nerve fiber layer; SCP, superficial capillary plexus; VD, vessel density; FD, VD within 300 μm width ring surrounding the foveal avascular zone.

## 4 Discussion

ASCVD includes a range of diseases primarily characterized by atherosclerosis, including CHD, ischemic stroke, and peripheral arterial disease. Recognized as a major global health threat (4–7), various regions have developed tools to assess ASCVD risk. Notably, the ASCVD risk calculator (2013) (9), created by the American College of Cardiology/American Heart Association (ACC/AHA), represents the first predictive model based on comprehensive data from White and Black American populations. The key variables include race (African American, White), gender, age, TC, HDL-C, systolic blood pressure (SBP), antihypertensive medication use, diabetes status, and smoking history. While the model offers satisfactory calibration for populations similar to its development cohort, its accuracy appears limited in other demographic groups (25–28). Furthermore, its predictive endpoints are confined to myocardial infarction and stroke. In 2014, the Joint British Societies (JBS) introduced the JBS3 risk score, a broader cardiovascular risk tool (11). This model extends its endpoints beyond myocardial infarction and stroke to include angina pectoris, coronary revascularization, transient ischemic attack, intermittent claudication, and related conditions. However, external validation of this tool is still pending. In 2016, China developed the Prediction for ASCVD Risk in China (China-PAR) score, which incorporated additional factors such as regional differences (north vs. south) and waist circumference. Its assessment endpoints included CHD-related mortality, myocardial infarction, and stroke (8). In comparison to the ASCVD risk calculator (2013) by the ACC/AHA, China-PAR demonstrates higher predictive accuracy for Chinese populations, but it has yet to be validated for non-Chinese adult cohorts.

Existing risk assessment tools are limited in their applicability across diverse populations and ethnicities. Moreover, these models are tailored to the general population, failing to address the specific needs of individuals with dyslipidemia, a group at heightened risk for ASCVD (1–3). In this study, significant differences in age and SBP were observed between the ASCVD and non-ASCVD groups, consistent with prior research in ASCVD risk models. However, no notable differences were found in HDL-C levels, diastolic blood pressure, or related parameters. Interestingly, TC and LDL-C levels were lower in the ASCVD group compared to the non-ASCVD group, likely reflecting the higher usage of lipid-lowering medications among ASCVD patients. These findings suggest that traditional indicators, such as TC, HDL-C, LDL-C, and DBP, may not be the most reliable markers for assessing ASCVD risk.

The retina, the only tissue with microvessels directly visible in the human body, has long been an important indicator for evaluating the effects of systemic conditions such as hypertension and diabetes on microvascular health (14, 21). Traditionally, color fundus photography is used to assess retinal blood vessels, primarily identifying signs like hemorrhage or neovascularization. The introduction of OCTA has advanced this assessment, offering a faster, non-invasive, and more detailed examination of retinal vascular changes (14, 29–31). Recent studies have established a relationship between retinal vascular parameters and coronary artery diseases. OCTA findings indicate that retinal VD is lower in patients with CHD compared to healthy controls (12, 32). Moreover, in CHD patients, higher Gensini scores correlate with reduced retinal VD (32). Patients with coronary total occlusion (CTO) also show higher Gensini scores and decreased retinal VD compared to those without CTO (33). The Gensini score, a standard clinical tool, assesses the severity of coronary artery stenosis (34). These observations suggest that retinal vasculature offers a distinct view of macrovascular changes. This study supports previous findings, noting that patients with ASCVD exhibit reduced retinal VD, including in the SCP and FD areas, alongside thinner RNFL and macular parafoveal thickness, when compared to individuals without ASCVD.

Among OCTA parameters, a reduction in retinal VD is indicative of vascular stenosis or occlusion. Existing literature confirms that hypertension, diabetes, and dyslipidemia contribute to decreases in retinal VD (16–18, 23, 35), with all three factors being well-established risks for ASCVD. In addition to ischemia caused by retinal vascular changes, RNFL and macular thickness are also influenced by local glucose levels and osmotic pressure (23).

In OCTA imaging, the detection region is divided into the macula and optic disc. Among the evaluated parameters, SCP perifovea VD, SCP parafovea VD, and FD correspond to macular superficial capillary plexus vessel densities and are correlated due to their interconnected capillary network. Combining these parameters results in a new composite measure, MSVD. Although DeLong test results did not demonstrate the superiority of MSVD over other indicators in assessing ASCVD risk in dyslipidemic patients, MSVD streamlines OCTA interpretation and provides practical benefits by enabling risk stratification based on macular scans alone, thus reducing both the scope of examination and associated costs. Considering its economic and clinical potential, MSVD represents a viable method for ASCVD screening in asymptomatic dyslipidemia patients. Its inclusion in future ASCVD risk prediction models merits further exploration.

The study has several limitations: (1) A cross-sectional design is initially selected for feasibility, but its statistical robustness in assessing the value of OCTA findings for evaluating ASCVD in individuals with dyslipidemia is inherently weaker than that of prospective cohort studies. (2) A higher proportion of patients in the ASCVD group are receiving medications (antihypertensive, lipid-lowering drugs, etc.) compared to the non-ASCVD group. Due to the small sample size, the study does not further stratify medication usage. (3) As a single-center study, it lacks the diversity of populations across different regions and ethnic groups, which limits the generalizability of the findings. Future research should involve a multicenter, multiracial prospective cohort study to adjust for confounding factors such as medication use and further assess the predictive value of retinal vascular changes detected by OCTA for ASCVD development in patients with dyslipidemia.

## 5 Conclusions

This study employs OCTA to assess retinal changes in dyslipidemia patients with ASCVD, identifying significant reductions in SCP perifovea VD, SCP parafovea VD, FD, RNFL, and macular thickness compared to those without ASCVD. These parameters may serve as potential biomarkers for evaluating ASCVD risk in individuals with dyslipidemia. Furthermore, a novel composite indicator, MSVD, is derived by combining the first three vessel density measures, offering an effective tool for ASCVD risk assessment in dyslipidemia patients. The incorporation of MSVD streamlines OCTA interpretation and lowers examination costs. OCTA-derived indicators, particularly MSVD, demonstrate considerable potential for integration into future ASCVD risk assessment models.

## Data Availability

The raw data supporting the conclusions of this article will be made available by the authors, without undue reservation.
